# Characterization of intrinsic molecular structure spectral profiles of feedstocks and co-products from canola bio-oil processing: impacted by source origin

**DOI:** 10.5713/ab.22.0077

**Published:** 2022-06-30

**Authors:** Alessandra M. R. C. B. de Oliveira, Peiqiang Yu

**Affiliations:** 1 Department of Animal and Poultry Science, College of Agriculture and Bioresources, University of Saskatchewan, Saskatoon, SK, S7N 5A8, Canada

**Keywords:** Canola Bio-oil Processing, Carbohydrate Structures, Co-Products, Feedstocks, Mid-Infrared, Molecular Spectroscopy, Molecular Structures, Protein 2nd Structures

## Abstract

**Objective:**

Feed molecular structures can affect its availability to gastrointestinal enzymes which impact its digestibility and absorption. The molecular spectroscopy-attenuated total reflectance Fourier transform infrared vibrational spectroscopy (ATR-FTIR) is an advanced technique that measures the absorbance of chemical functional groups on the infrared region so that we can identify and quantify molecules and functional groups in a feed. The program aimed to reveal the association of intrinsic molecular structure with nutrient supply to animals from canola feedstocks and co-products from bio-oil processing. The objective of this study was to characterize special intrinsic carbohydrate and protein-related molecular structure spectral profiles of feedstock and co-products (meal and pellets) from bio-oil processing from two source origins: Canada (CA) and China (CH).

**Methods:**

The samples of feedstock and co-products were obtained from five different companies in each country arranged by the Canola Council of Canada (CCC). The molecular structure spectral features were analyzed using advanced vibrational molecular spectroscopy-ATR-FTIR. The spectral features that accessed included: i) protein-related spectral features (Amide I, Amide II, α-helix, β-sheet, and their spectral intensity ratios), ii) carbohydrate-related spectral features (TC1, TC2, TC3, TC4, CEC, STC1, STC2, STC3, STC4, TC, and their spectral intensity ratios).

**Results:**

The results showed that significant differences were observed on all vibrationally spectral features related to total carbohydrates, structural carbohydrates, and cellulosic compounds (p<0.05), except spectral features of TC2 and STC1 (p>0.05) of co-products, where CH meals presented higher peaks of these structures than CA. Similarly, it was for the carbohydrate-related molecular structure of canola seeds where the difference between CA and CH occurred except for STC3 height, CEC and STC areas (p>0.05). The protein-related molecular structures were similar for the canola seeds from both countries. However, CH meals presented higher peaks of amide I, α-helix, and β-sheet heights, α-helix:β-sheet ratio, total amide and amide I areas (p<0.05).

**Conclusion:**

The principal component analysis was able to explain over 90% of the variabilities in the carbohydrate and protein structures although it was not able to separate the samples from the two countries, indicating feedstock and coproducts interrelationship between CH and CA.

## INTRODUCTION

Canola (*Brassica napus*) has been extensively produced in Canada since its development in 1970s. Canola was developed due the necessity for an oilseed with high amounts of oil that could be extracted for human consumption that was palatable (low erucic acid levels) and the co-product could be utilized to minimize waste (low glucosinolates levels increases palatability of the meal for animal consumption). Therefore, canola is the oil rapeseed that resulted from extensive studies of plant breeding and selection at the University of Manitoba by Dr. Stefansson and his team in 1974 [1].

Regular wet laboratory analyses determine the chemical composition of feedstuffs but fail to characterize their carbohydrate and protein structures, meaning they do not provide information related to the real nutrient supply and utilization that are essential for animal performance [2,3]. Plus, the use of harsh chemicals for wet chemistry analyses can alter and destroy these structures consequently over or underestimating results [4].

Spectroscopy studies the interaction of light and matter and provides information on the chemical composition and physical structures at specific locations of a sample through imaging techniques [5,6]. A quick and non-invasive method of analysis that observes the mid-infrared region (ca. 4,000 to 800 cm^−1^) called attenuated total reflectance Fourier transform infrared vibrational spectroscopy (ATR-FTIR) identifies molecules and functional groups based on their infrared light absorbance on this region [3].

The structure of protein in a matter is essential to gain knowledge about its availability to the animals. For instance, protein is stored in seeds as cruciferin or napin. Perera et al [7] mentioned that 60% of *B. napus’s* protein storage is as cruciferin and only 20% is as napin. The napin fraction contains 40% to 46% of α-helix and lower amounts of β-sheet, while the cruciferin contains about 10% of α-helix and around 50% of β-sheet. High amounts of β-sheet indicate a low protein value because this structure provides low availability to the gastrointestinal enzymes [8,9]. Therefore, identifying the presence of these structures on feedstuffs is essential to understand how the animal can respond when fed.

The use of ATR-FTIR to characterize the carbohydrate and protein structures in feedstuffs is important to provide data to increase the knowledge of these structures on these materials and to identify structural variations due to transport and processing methods. Furthermore, the aim of this study was to identify the intrinsic carbohydrate and protein structures of canola seeds and meals from five crushing companies in Canada and five in China.

## MATERIALS AND METHODS

### Sampling and analyses

The samples of feedstocks and co-products from bio-oil processing were collected from Canada and China by the Canola Council of Canada. Samples were collected from five crusher companies operating in China and five in Canada. The companies crushed seeds which were imported from Canada. Three of the five Canadian crushers samples of meals were pelleted and two were mash, like China’s meals that were all mash. Samples of seeds and meals were collected from different batches from each crusher, stored and transported to the University of Saskatchewan in Canada for further analyses. The samples were provided by each company’s quality control laboratory and are to be considered representative of the reality of those crushers.

### Attenuated total reflectance-fourier transform infrared vibrational molecular spectroscopy

The spectral analyses of the intrinsic molecular structures of protein and carbohydrate of the canola seeds, meals and pellets were obtained at the University of Saskatchewan, using the FTIR-ATR vibrational spectroscopy model 4200 (JASCO Corporation, Tokyo, Japan) machine at the mid-infrared spectrum (ca. 4,000 to 800 cm^−1^).

With the assistance of the OMNIC 7.3 software (Spectra Tech., Madison, WI, USA), the spectra were represented in images, and transcribed into numbers, so they later could be processed by Unscrambler X 10.3 (CAMO Software, 2013) for the multivariate analyses.

### Statistical analysis

Fitting a complete randomized block design (RCBD), with country and company as fixed effects and batch as random effect, the procedure MIXED was used on SAS 9.4 (SAS Institute, USA).


$$y=\mu +\tau_{i}+\beta_{j}+{ɛ}_{ij}$$

Where, *μ* = overall mean; τ_*i*_ = fixed effect; *β* = random effect; *β*_*ij*_ = error. *β*_*j*_ ~ NIID (normally, identically, and independently distributed) *β*_*ij*_ ~ NIID (normally, identically, and independently distributed). When p<0.05 results were considered significant. The multiple comparison was tested through the Tukey method.

## RESULTS AND DISCUSSION

The intrinsic molecular carbohydrate structures of canola meals and seeds are shown in Table 1 and 2. This study analyzed the heights of four total carbohydrate peaks (TC1, TC2, TC3, and TC4) as well as the cellulosic compounds (CEC), and four of the structural carbohydrates (STC1, STC2, STC3, and STC4), and the areas of total carbohydrate, CECs, and STCs.

The analyses within Canada showed differences between samples that were pelleted and mash for most structures studied (p<0.05), except for STC1 and STC2 that presented differences among companies (p<0.05) but no differences between pellets or mash (p>0.05), and the area of CEC that was same among the five Canadian crushers (p>0.05) (Table 1). Amidst the Chinese crushers, only TC4 height varied (p = 0.040). Interestingly for the CHO structures studied on this project, almost all were in higher concentration of the Chinese meals, except for TC2 and STC1 that were the same between both countries (p = 0.057 and p = 0.700, respectively).

The seeds were more even in general within and between countries than the meals (Table 2). There were no differences within the crushing companies in China (p>0.05), and only STC1 and STC3 showed some differences among the Canadian crushers (p = 0.009 and p = 0.044, respectively). Like the meals, most parameters were different between countries (p<0.05) apart from STC3 height (p = 0.100), and the areas of CEC (p = 0.804) and STC (p = 0.284). Opposite from the meals, the seeds presented higher carbohydrate structures concentrated on the seeds from Canadian crushers.

The inherent protein structures of canola meals and seeds are displayed in Tables 3 and 4. Table 3 shows that only the heights of the α-helix and β-sheet were higher in the pelleted meals than the mash (p = 0.028 and p = 0.032, respectively). Similar to the carbohydrate structures, the protein structures of the canola meals from Chinese crusher were all different among themselves (p<0.05). Chinese samples presented higher Amide I height (p = 0.011), α-helix height (p = 0.001), β-sheet height (p = 0.012), α-helix to β-sheet ratio (p = 0.008), Amide area (p = 0.038), and Amide I area (p = 0.019) than the Canadian meals. Despite the variations observed on the protein structures of the meals, the seeds did not result in any differences between countries or within Chinese crushers (p>0.05). Only the α-helix to β-sheet ratio of Canadian seeds varied among companies (p<0.001).

Industry processing methods can affect protein structures [8–11]. It was observed that the α-helix:β-sheet ratio decreased from the canola seeds to canola meals (1.07 vs 1.02, respective averages between countries), indicating that the oil extraction process increased the amount of β-sheet that is related to low availability of the protein (Tables 3, 4).

Theodoridou and Yu [8–11] found α-helix:β-sheet ratio of 0.96 and Amide I:Amide II area ratio of 2.70 for canola meals. These results were close but lower than ours of 1.02 and 3.01, respectively (Table 3). Ban et al [2], using Synchrontron FTIR, reported a new line of brown canola seeds with α-helix: β-sheet ratio of 1.24 and Amide I:Amide II area ratio of 2.46. The canola seeds analyzed on our study showed lower α-helix:β-sheet ratio (1.07) and higher Amide I:Amide II area ratio (3.14). And Ban et al [2] reported canola seeds having Amide I:Amide II height ratio of 1.73 (lower than ours 2.23) and α-helix:β-sheet ratio of 1.06. Although some variations are observed our results are in range with the literature.

**Figure 1:**
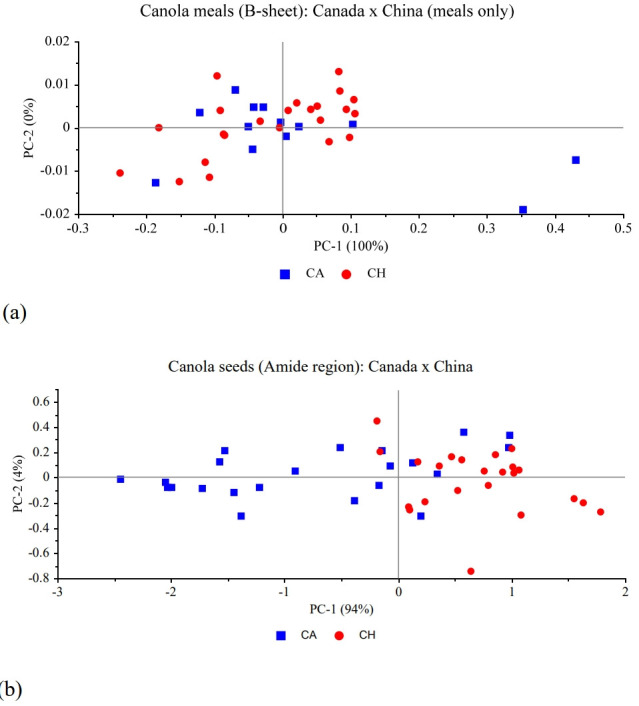
Principal component analysis (PCA) of (a) β-sheet region from canola meals, (b) Amide region from canola meals: comparison between Canada and China.

**Figure 2:**
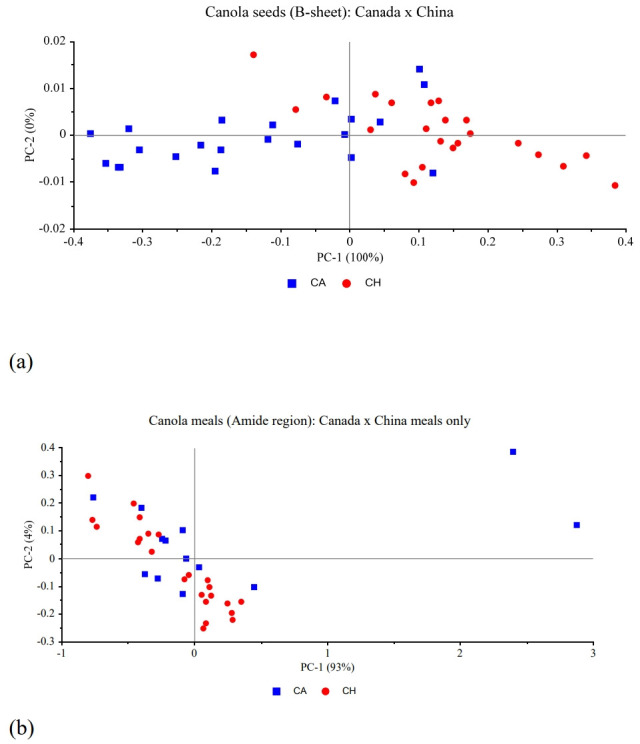
Principal Component Analysis (PCA) of (a) β-sheet region from canola seeds, (b) Amide region from canola seed: comparison between Canada and China.

The principal component analysis (PCA) of a few carbo hydrate and protein related structures of canola seeds and meals are presented from Figures 1 and 2 comparing Canada and China. The first principal component was able explain from 93% to 100% of the variability in the samples of the structures represented here and it was not possible to completely distinguish the samples from the two countries. Therefore, both seeds and meals are not completely different regarding the carbohydrate or protein spectral features between Canada and China.

## CONCLUSION

The FTIR-ATR analysis on the carbohydrate-related features showed a significant difference between Canadian companies and between the meals and pellets. Although not different within companies, when compared to Canada, the Chinese meals showed higher peak heights for total carbohydrate (TC3, TC4), CEC, structural carbohydrates (STC2, STC3, and STC4), and areas for TC, CEC, and STC (p<0.05). Canadian canola seeds presented higher peaks for TC1, TC2, TC3, TC4, CEC, STC2, STC4, and TC area, while the ones from China showed a higher peak for SCT1 (p = 0.033).

The FTIR-ATR analysis of the protein-related structures of canola seeds showed no differences between countries, and only the α-helix:β-sheet ratio was different among the Canadian companies. The Chinese meals, however, were all different between each other. Amide I height; α-helix and β-sheet heights and their ratio; and amide and amide I areas; were all higher in Chinese meals than Canadian meals and pellets. The PCA reported showed the comparisons of some protein and some carbohydrate-related aspects of canola meals and seeds and it was not possible to completely differentiate the protein or the carbohydrate structures between countries.

In conclusion, the FTIR-ATR is able detect the structural characteristics of canola meal and canola seeds that are related to nutrient profiles. The PCA was able to highly explain over 90% of the variabilities in the carbohydrate and protein structures and was not able to separate the samples from the two countries, indicating feedstock and coproducts interrelationship between CH and CA.

## Figures and Tables

**Table 1:** Using FTIR-ATR molecular spectroscopic technique to determine carbohydrates-related molecular spectral features of canola meals and pellets: comparisons between companies, countries, and periods

Items Unit: AU	Height									Area		

	Total carbohydrate peaks (TC)				Cellulosic compounds peak (CEC)	Structural carbohydrates (ST) peaks				TC peak	CEC peak	STC peak
	
	Peak 1 (TC1)	Peak 2 (TC2)	Peak 3 (TC3)	Peak 4 (TC4)		Peak 1 (STC1)	Peak 2 (STC2)	Peak 3 (STC3)	Peak 4 (STC4)			
Canadian processing plants												
Plant 1 (M)	0.48^b^	0.46^b^	0.35	0.16^b^	0.06	0.06^a^	0.06^a^	0.11	0.06	76.18^b^	3.80	18.24^ab^
Plant 2 (M)	0.51^ab^	0.49^ab^	0.37	0.19^ab^	0.06	0.03^b^	0.05^b^	0.11	0.12	81.56^ab^	3.24	16.51^b^
Plant 3 (P)	0.55^a^	0.52^a^	0.39	0.20^a^	0.07	0.04^ab^	0.06^ab^	0.12	0.11	86.49^a^	3.53	18.26^ab^
Plant 4 (P)	0.51^ab^	0.49^ab^	0.38	0.19^a^	0.07	0.04^ab^	0.06^a^	0.12	0.13	82.30^ab^	3.51	18.58^a^
Plant 5 (P)	0.51^ab^	0.49^ab^	0.37	0.18^ab^	0.07	0.04^ab^	0.06^a^	0.11	0.13	80.94^ab^	3.56	18.23^ab^
SEM	0.011	0.010	0.010	0.010	0.003	0.005	0.002	0.005	0.019	2.010	0.141	0.460
p-value	0.010	0.041	0.047	0.028	0.253	0.041	0.013	0.204	0.071	0.022	0.096	0.032
Meals vs pellets												
SEM	0.061	0.058	0.054	0.050	0.015	0.027	0.011	0.023	0.097	10.521	0.739	2.310
p-value	0.027	0.041	0.016	0.023	0.038	0.324	0.052	0.028	0.047	0.023	0.974	0.022
Chinese processing plants												
Plant A	0.51	0.49	0.38	0.19^b^	0.08	0.05	0.06	0.11	0.13	82.08	3.94	19.48
Plant B	0.52	0.49	0.38	0.19^ab^	0.07	0.05	0.07	0.12	0.12	83.56	3.94	20.55
Plant C	0.52	0.49	0.38	0.20^a^	0.07	0.04	0.06	0.12	0.12	84.48	3.82	19.13
Plant D	0.52	0.49	0.38	0.19^ab^	0.07	0.05	0.07	0.12	0.12	83.61	3.86	19.98
Plant E	0.52	0.48	0.37	0.19^ab^	0.07	0.05	0.07	0.12	0.12	82.79	3.77	19.88
SEM	0.006	0.007	0.004	0.003	0.002	0.003	0.003	0.002	0.003	0.828	0.150	0.472
p-value	0.377	0.603	0.250	0.040	0.258	0.194	0.165	0.101	0.281	0.288	0.834	0.250
Overall												
CA Plants	0.50	0.47	0.35	0.17	0.06	0.05	0.06	0.11	0.08	78.57	3.56	17.47
CH Plants	0.52	0.49	0.38	0.19	0.07	0.05	0.06	0.12	0.12	83.35	3.83	19.73
SEM	0.007	0.007	0.006	0.006	0.002	0.004	0.002	0.003	0.011	1.182	0.121	0.403
p-value	0.011	0.057	0.005	0.001	<0.001	0.700	<0.001	0.002	0.006	0.002	0.023	<0.001

FTIR-ATR, Fourier transform infrared-attenuated total reflectance; TC, total carbohydrate; STC, structural carbohydrate; CEC, cellulosic compound; 1, 2, 3 and 4: correspond to the different peaks; SEM, standard error of the mean; CA, Canada; CH, China; M, meals; P, meals pelleted; Overall, compares only meals.

a,b Means in the same column with different letters differ significantly (p<0.05).

**Table 2:** Using FTIR-ATR molecular spectroscopic technique to determine carbohydrates-related molecular spectral features of canola seeds: comparisons between companies, countries, and periods

Items Unit: AU	Height									Area		

	Total carbohydrate peaks (TC)				Cellulosic compounds peak (CEC)	Structural carbohydrates (ST) peaks				TC peak	CEC peak	STC peak
	
	Peak 1 (TC1)	Peak 2 (TC2)	Peak 3 (TC3)	Peak 4 (TC4)		Peak 1 (STC1)	Peak 2 (STC2)	Peak 3 (STC3)	Peak 4 (STC4)			
Canadian processing plants												
Plant 1	0.55	0.49	0.36	0.19	0.07	0.07^b^	0.07	0.10^b^	0.11	84.06	3.69	20.60
Plant 2	0.55	0.53	0.37	0.18	0.07	0.09^ab^	0.08	0.12^ab^	0.03	87.67	4.36	22.77
Plant 3	0.56	0.51	0.37	0.16	0.07	0.09^ab^	0.08	0.12^ab^	0.03	85.98	4.25	23.23
Plant 4	0.55	0.51	0.37	0.16	0.07	0.10^a^	0.09	0.12^ab^	0.04	84.26	4.27	24.49
Plant 5	0.53	0.51	0.37	0.16	0.07	0.10^a^	0.09	0.13^a^	0.02	83.20	4.35	24.94
SEM	0.244	0.214	0.019	0.018	0.005	0.008	0.005	0.007	0.033	3.906	0.246	1.281
p-value	0.915	0.655	0.981	0.664	0.954	0.009	0.050	0.044	0.189	0.873	0.225	0.075
Chinese processing plants												
Plant A	0.50	0.48	0.33	0.12	0.06	0.10	0.08	0.11	0.00	77.15	4.11	22.21
Plant B	0.51	0.48	0.34	0.14	0.06	0.10	0.09	0.12	0.00	78.63	4.10	22.86
Plant C	0.51	0.49	0.35	0.14	0.07	0.10	0.08	0.12	0.01	79.69	4.13	22.41
Plant D	0.49	0.48	0.33	0.13	0.07	0.10	0.08	0.12	0.00	76.94	4.29	22.44
Plant E	0.48	0.47	0.32	0.14	0.06	0.10	0.08	0.12	0.00	74.16	4.03	21.78
SEM	0.012	0.011	0.010	0.009	0.002	0.003	0.003	0.003	0.012	1.838	0.100	0.707
p-value	0.414	0.503	0.283	0.531	0.582	0.747	0.939	0.547	0.657	0.221	0.377	0.800
Overall												
CA Plants	0.55	0.51	0.37	0.17	0.07	0.09	0.08	0.12	0.05	84.81	4.16	23.08
CH Plants	0.50	0.48	0.34	0.14	0.06	0.10	0.08	0.12	0.00	77.59	4.14	22.40
SEM	0.010	0.010	0.009	0.007	0.001	0.003	0.002	0.003	0.011	1.737	0.083	0.592
p-value	<0.001	0.005	<0.001	<0.001	<0.004	0.033	0.044	0.100	<0.001	<0.001	0.804	0.284

FTIR-ATR, Fourier transform infrared-attenuated total reflectance; TC, total carbohydrate; STC, structural carbohydrate; CEC, cellulosic compound; 1, 2, 3 and 4: correspond to the different peaks; SEM, standard error of the mean; CA, Canada; CH, China.

a,b Means in the same column with different letters differ significantly (p<0.05).

**Table 3:** Using FTIR-ATR molecular spectroscopic technique to determine protein related molecular spectral features of canola meals and pellets: comparisons between companies, countries, and periods

Items Unit: AU	Height		Ratio	Height		Ratio	Area			Ratio
					
	Amide I	Amide II	Amide I: Amide II	α-helix	β-sheet	α-helix: β-sheet	Amide	Amide I	Amide II	Amide I: Amide II
Canadian processing plants										
Plant 1 (M)	0.35	0.18	1.99	0.32	0.32	0.99	50.76	27.02	9.04	3.08
Plant 2 (M)	0.38	0.21	1.88	0.35	0.34	1.00	53.36	28.46	10.25	2.93
Plant 3 (P)	0.39	0.22	1.93	0.37	0.36	1.00	55.50	29.42	10.45	3.01
Plant 4 (P)	0.40	0.22	1.93	0.38	0.37	1.02	57.19	30.56	10.90	2.93
Plant 5 (P)	0.39	0.23	1.78	0.37	0.35	1.03	54.64	29.24	10.94	2.79
SEM	0.025	0.030	0.140	0.031	0.027	0.022	2.320	2.797	1.769	0.205
p-value	0.193	0.283	0.481	0.124	0.132	0.538	0.236	0.209	0.422	0.664
Meals vs pellets										
SEM	0.082	0.075	0.405	0.091	0.072	0.112	11.14	5.830	4.237	0.729
p-value	0.052	0.084	0.451	0.028	0.032	0.226	0.066	0.062	0.140	0.453
Chinese processing plants										
Plant A (M)	0.40^a^	0.22^a^	1.85^b^	0.37^ab^	0.37^a^	1.01^b^	54.08^ab^	29.79^ab^	10.86^a^	2.85^b^
Plant B (M)	0.41^a^	0.22^a^	1.92^ab^	0.38^a^	0.37^a^	1.02^b^	56.74^a^	31.19^a^	10.98^a^	2.99^ab^
Plant C (M)	0.41^a^	0.21^a^	2.02^a^	0.39^a^	0.37^a^	1.05^ab^	55.84^a^	30.97^a^	10.96^a^	2.98^ab^
Plant D (M)	0.41^a^	0.21^a^	1.99^a^	0.39^a^	0.36^a^	1.08^a^	56.48^a^	30.93^a^	10.71^a^	3.03^a^
Plant E (M)	0.37^b^	0.20^b^	1.91^ab^	0.35^b^	0.34^b^	1.05^ab^	51.86^b^	28.40^b^	10.06^b^	2.99^a^
SEM	0.018	0.026	0.152	0.021	0.025	0.019	1.605	2.620	1.758	0.221
p-value	0.001	<0.001	0.013	<0.001	<0.001	0.014	0.001	0.001	0.004	0.017
Overall										
CA Plants	0.37	0.20	1.94	0.33	0.33	0.99	51.97	27.71	9.59	3.01
CH Plants	0.40	0.21	1.97	0.37	0.36	1.04	55.16	29.59	10.31	3.01
SEM	0.017	0.023	0.117	0.021	0.022	0.015	1.629	2.346	1.473	0.171
p-value	0.011	0.175	0.585	0.001	0.012	0.008	0.038	0.019	0.149	0.977

FTIR-ATR, Fourier transform infrared-attenuated total reflectance; SEM, standard error of the mean; CA, Canada; CH, China; M, meals; P, meals pelleted; Overall, compares only meals.

a,b Means in the same column with different letters differ significantly (p<0.05).

**Table 4:** Using FTIR-ATR molecular spectroscopic technique to determine protein related molecular spectral features of canola seeds: comparisons between companies, countries, and periods

Items Unit: AU	Height		Ratio	Height		Ratio	Area			Ratio
					
	Amide I	Amide II	Amide I: Amide II	α-helix	β-sheet	α-helix: β-sheet	Amide	Amide I	Amide II	Amide I: Amide II
Canadian processing plants										
Plant 1	0.33	0.16	2.19	0.33	0.30	1.10^ab^	42.50	23.32	8.31	3.04
Plant 2	0.32	0.16	2.02	0.31	0.28	1.12^a^	41.66	22.74	8.92	2.72
Plant 3	0.31	0.15	2.16	0.28	0.27	1.05^bc^	41.11	21.72	7.80	3.10
Plant 4	0.33	0.15	2.36	0.31	0.31	1.01^c^	42.84	23.86	7.91	3.37
Plant 5	0.34	0.15	2.37	0.32	0.30	1.06^abc^	46.81	24.58	8.68	3.18
SEM	0.014	0.014	0.161	0.017	0.015	0.016	2.266	1.550	1.339	0.356
p-value	0.533	0.471	0.067	0.234	0.229	<0.001	0.294	0.365	0.385	0.255
Chinese processing plants										
Plant A	0.29	0.14	2.22	0.27	0.26	1.05	39.62	21.39	7.79	3.13
Plant B	0.31	0.14	2.42	0.29	0.28	1.06	43.21	22.98	7.98	3.42
Plant C	0.33	0.16	2.24	0.32	0.30	1.08	45.52	24.88	8.85	3.20
Plant D	0.33	0.16	2.14	0.31	0.29	1.08	43.28	23.82	9.18	2.81
Plant E	0.35	0.16	2.35	0.33	0.31	1.07	48.08	25.91	9.04	3.17
SEM	0.017	0.017	0.212	0.021	0.018	0.024	3.392	1.983	1.598	0.456
p-value	0.135	0.100	0.414	0.136	0.083	0.736	0.248	0.205	0.152	0.607
Overall										
CA Plants	0.33	0.15	2.23	0.31	0.29	1.07	42.68	23.23	8.22	3.14
CH Plants	0.33	0.15	2.28	0.31	0.29	1.07	44.79	23.87	8.38	3.17
SEM	0.009	0.013	0.151	0.014	0.014	0.010	1.752	1.358	1.311	0.317
p-value	0.894	0.532	0.471	0.962	0.688	0.988	0.186	0.433	0.610	0.839

FTIR-ATR, Fourier transform infrared-attenuated total reflectance; SEM, standard error of the mean; CA, Canada; CH, China.

a–c Means in the same column with different letters differ significantly (p<0.05).
